# A Machine Learning Approach Using Topic Modeling to Identify and Assess Experiences of Patients With Colorectal Cancer: Explorative Study

**DOI:** 10.2196/58834

**Published:** 2025-01-27

**Authors:** Kelly Voigt, Yingtao Sun, Ayush Patandin, Johanna Hendriks, Richard Hendrik Goossens, Cornelis Verhoef, Olga Husson, Dirk Grünhagen, Jiwon Jung

**Affiliations:** 1 Department of Surgical Oncology and Gastrointestinal Surgery, Erasmus Medical Centre Cancer Institute, Rotterdam, Netherlands; 2 Faculty of Industrial Design Engineering, Delft University of Technology, Delft, Netherlands; 3 Faculty of Electrical Engineering, Mathematics and Computer Science, Delft University of Technology, Delft, Netherlands; 4 Department of Surgery, Erasmus Medical Centre, Rotterdam, Netherlands; 5 Department of Medical Oncology, Netherlands Cancer Institute, Amsterdam, Netherlands

**Keywords:** colorectal cancer, forum, topic modeling, patient journey, patient experience, AI, machine learning, cancer care, cancer survivor, United States, quality of life, post, topic, artificial intelligence

## Abstract

**Background:**

The rising number of cancer survivors and the shortage of health care professionals challenge the accessibility of cancer care. Health technologies are necessary for sustaining optimal patient journeys. To understand individuals’ daily lives during their patient journey, qualitative studies are crucial. However, not all patients wish to share their stories with researchers.

**Objective:**

This study aims to identify and assess patient experiences on a large scale using a novel machine learning–supported approach, leveraging data from patient forums.

**Methods:**

Forum posts of patients with colorectal cancer (CRC) from the Cancer Survivors Network USA were used as the data source. Topic modeling, as a part of machine learning, was used to recognize the topic patterns in the posts. Researchers read the most relevant 50 posts on each topic, dividing them into “home” or “hospital” contexts. A patient community journey map, derived from patients stories, was developed to visually illustrate our findings. CRC medical doctors and a quality-of-life expert evaluated the identified topics of patient experience and the map.

**Results:**

Based on 212,107 posts, 37 topics and 10 upper clusters were produced. Dominant clusters included “Daily activities while living with CRC” (38,782, 18.3%) and “Understanding treatment including alternatives and adjuvant therapy” (31,577, 14.9%). Topics related to the home context had more emotional content compared with the hospital context. The patient community journey map was constructed based on these findings.

**Conclusions:**

Our study highlighted the diverse concerns and experiences of patients with CRC. The more emotional content in home context discussions underscores the personal impact of CRC beyond clinical settings. Based on our study, we found that a machine learning-supported approach is a promising solution to analyze patients’ experiences. The innovative application of patient community journey mapping provides a unique perspective into the challenges in patients’ daily lives, which is essential for delivering appropriate support at the right moment.

## Introduction

Colorectal cancer (CRC), the third most common cancer in the Netherlands significantly impacts the health of individuals [1]. Supportive care involves adopting a person-centered care approach, intending to offer individuals affected by cancer the essential services required to address their informational, emotional, social, and physical needs and concerns throughout the entire patient journey [2,3]. Understanding the needs and concerns of those affected by this disease during their patient journey is crucial for improving patient outcomes and quality of care [4]. Several survey and qualitative studies involving CRC survivors offer valuable information about the needs and perceptions of patients [4-7]. However, these survey- and qualitative studies are limited by sample size, labor-intensive processes, and could lead to socially desirable answers [8]. Efforts were made to include vulnerable populations who may not be accessible through focus groups, ensuring a more comprehensive understanding of diverse patient perspectives [9]. In the last decade, patient web forums have emerged as valuable platforms for individuals to openly share their experiences and thoughts related to CRC, providing unique insights into the social, physical, and emotional aspects of their patient journey [10,11]. These forums offer different opinions compared with traditional patient experience data collection methods, such as questionnaires and interviews.

While patient community forums contain a wealth of information, the analysis of the extensive unstructured data within these forums poses a considerable challenge [12]. Traditional manual qualitative analysis by human experts is time-consuming, labor-intensive, and lacks scalability, making it impractical to analyze the sheer volume of patient-generated unstructured content. Machine learning techniques offer a potential solution to address this challenge. This enables the automated processing and analysis of textual data, allowing for the efficient extraction and interpretation of large amounts of patient forum posts [13,14].

Our primary objective is to assess patient experiences using a novel machine learning–supported approach and data from patient forums. To achieve this, we used patient community journey mapping to better understand the experiences throughout the patient journey [15]. This is a machine learning-driven approach, that uses web-based patient forums as input data and is processed through topic modeling. By gaining insights into these patient experiences, we can shape future patient journeys such as remote monitoring systems to be aligned with current patients’ needs.

## Methods

### Ethical Considerations

Ethical approval was given by the human research ethics committee of the Delft University of Technology (ID 2596). Informed consent was not required since the data used in this study were sourced from publicly available forums, in accordance with institutional guidelines. The data were accessed and analyzed in accordance with the terms of service and privacy policies outlined by the platform hosting the data. As the ethics committee suggested, all data used in this study were anonymized by removing all direct and indirect identifiers (eg, names, location, and user ID) to prevent potential privacy issues within our data set. Confidentiality was strictly maintained during data collection, analysis, and reporting to ensure that no identifiable information was disclosed.

In order to assess patient experiences using machine learning–driven analysis, this study used systematic data collection, advanced topic modeling techniqu and cocreation sessions with domain experts to interpret and validate identified clusters. The Consolidated Reporting Guidelines for prognostic and diagnostic machine learning modeling studies checklist can be found in Multimedia Appendix 1.

### Data Source

To enable research, we used forum posts of patients with CRC scraped from the Cancer Survivors Network USA, an open-source patient community platform. The web-based platform provides support, education, and advocacy for patients affected by CRC that includes current patients, former patients, families, and caregivers. The posts are written with the intention of asking questions to peers and health care providers, as well as forsharing experiences among peers [16]. The initial CRC discussion thread (ie, main posts, comments, and replies) has remained active on the public platform since the year 2000. No distinctions were made between main thread posts and replies to posts.

The data collection involves 2 main steps: first involves using Selenium WebDriver to gather the URLs of discussion pages, and second, using BeautifulSoup to extract data from the HTML elements while keeping sensitive information secure.

### Data Analysis (Topic Modeling)

Information about the personal (health) status of patients, which could be directly or indirectly identifiable, was excluded from the analysis. Topic modeling, a machine learning algorithm was used to recognize patterns in the platform data. Nonnegative matrix factorization (NMF) was used as a topic modeling technique to analyze the data set and identify topics with weighted keywords [17,18]. The number of topics was determined by evaluating topic coherence scores and model stability [19,20]. Model stability was assessed using Jaccard similarity to reduce the overlap of topics. Human evaluation was used to ensure that the output contains diverse and distinctive topics, without topics unrelated to patient experiences. This evaluation was conducted by 4 researchers to select the number of topics that yield the most diverse and distinctive clusters.

### Interpretation of Data Analysis (Qualitative Analysis)

To comprehend each topic, researchers meticulously read and analyzed the top 50 most relevant posts and top 20 related keywords. The most relevant posts and keywords had the highest dominant topic score identified by the algorithm, indicating they were most closely associated with the topic. The algorithm efficiently identified the most relevant posts and keywords within each topic by using Term Frequency–Inverse Document Frequency to calculate the importance of each term in relation to each document [21].

### Clusters

The identified topics were grouped into clusters for topics that revolved around similar concerns. Irrelevant topics (eg, those related to platform-specific issues) were excluded. The frequency of discussions on each topic was recorded by counting the number of posts associated with each topic.

### Creation of Patient Community Journey Map

This study uses patient community journey mapping to visually present the identified patient experience topics. The creation of the patient community journey map began with the conduction of 2 interviews with CRC medical doctors to outline the patient journey and its distinct phases such as diagnosis and follow-up of a patient with CRC.

The results of the interpreted topics were deliberated in an in-person cocreation session involving domain experts [22]. The cocreation session took place in the Erasmus Medical Centre in March 2023 and lasted 3 hours. The interdisciplinary team of domain experts reviewed and discussed the outputs comprehensively. The team comprised 2 oncological surgeons, 2 surgical oncology PhD candidates (MD), and an epidemiologist with an interest in oncological research. A total of 3 out of 5 participants have over 20 years of work experience, indicating a high level of expertise. During the cocreation session, clusters from the NMF results were interpreted, and feedback was provided on the generated interpretations. This process ensured a thorough examination and validation of the identified clusters within a clinically relevant context. During this session, the same domain experts also reviewed the patient community journey map. The identified topics were positioned on the journey map, with allocation to specific phases of the patient journey. Distinctions were made regarding whether each topic was taking place at a “hospital” or “home”setting.

## Results

### Data

A total of 294,166 posts were extracted from the patient forums of the Cancer Survivors Network USA. The posts on the website were created between the year 2000 and 2022. However, 212,107 posts were analyzed, as the remaining 82,059 posts were excluded through topic modeling.

### Topic Modeling

Using NMF topic modeling, the topic coherence score, and model stability did not provide clear insights as the coherence score consistently declined. Therefore, human evaluation was required. Initially, 40 and 50 topics were considered the best amount of topics. Subsequently, the cut-off of 45 topics was also reviewed. Ultimately, 50 topics were chosen as the number that provided the most diverse and distinctive topics. This was confirmed by examining keywords and the most relevant posts for each cluster. Additionally, one topic bin identified 82,059 posts as unclassifiable," which could not be categorized into any of the 50 topics.

### Identified Patient Experience Topics and Patient Community Journey Map

A total of 50 topics were identified with the use of NMF topic modeling. Thirteen topics were excluded as they were unrelated to patient experiences, such as platform use and expressions of gratitude. The data export of the relevant topics is shown in Table 1. These are the key patient experiences found in our study.

**Figure 1.**
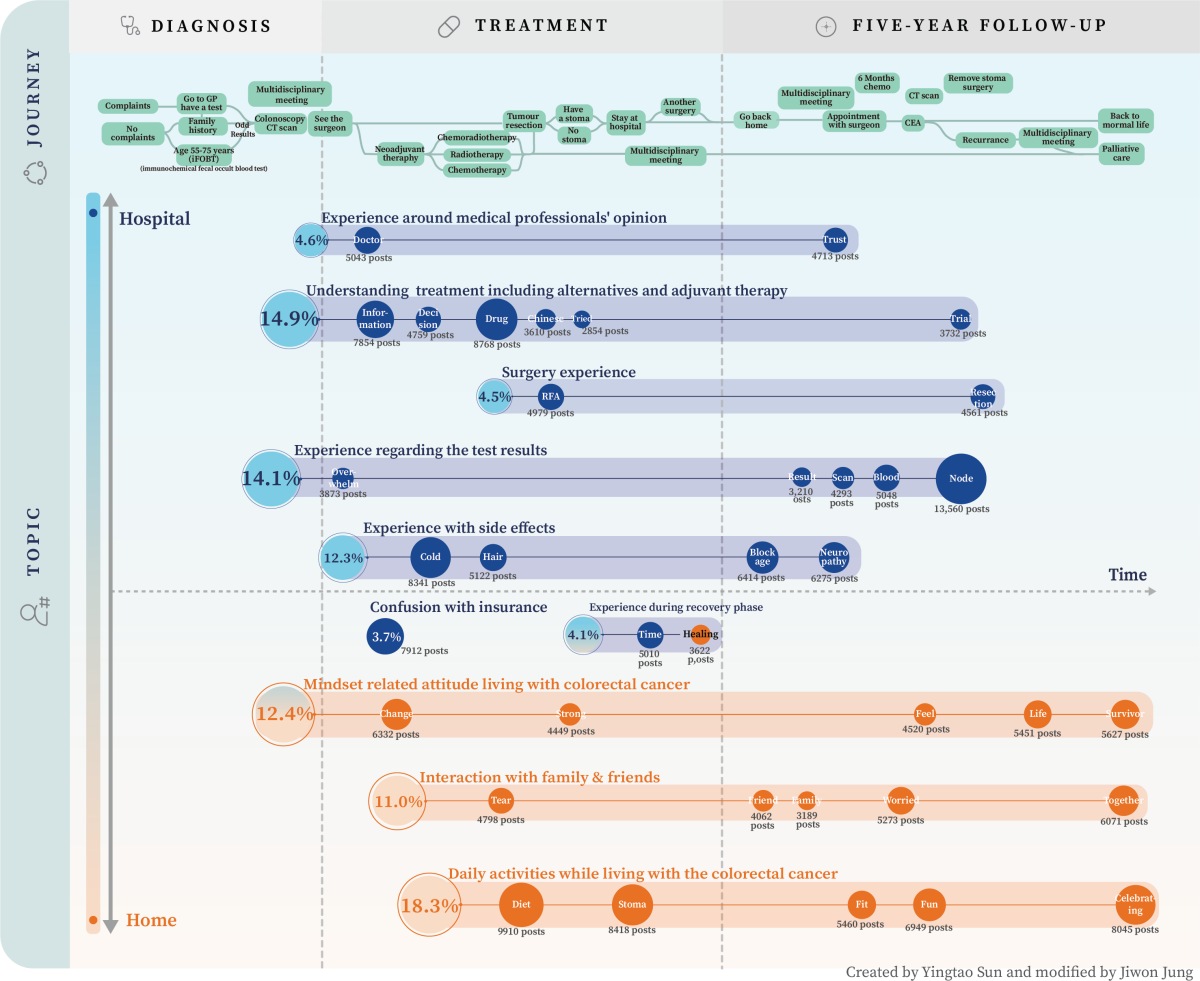
Community journey map of patients with colorectal cancer. CEA: carcinoembryonic antigen; CT: computed tomography; GP: general practitioner; RFA: radiofrequency ablation.

A total of 10 clusters were derived from 37 topics. The patient community journey map, shown in Figure 1, serves as a visual guide to navigate through the nuanced dynamics of patient experiences.

The topics in the home context have a more emotional content, as emotionally charged keywords such as “confused,” “bad,”and “worried” are more often discussed. Conversely, the hospital context is marked by the clinical terminologies “drug” and “node.” This reflects the distinct atmospheres characterizing discussions in different contexts.

The clusters commanding the highest share of posts are “Daily activities while living with CRC” (38,782, 18.3%) and, “Understanding treatment including alternatives and adjuvant therapy” (31,577, 14.9%) underscoring their important role in shaping the experiences of CRC survivors. Patients expressed significant concerns about test results, as indicated by the 14.1% of posts within cluster 4. As shown in Figure 1, “Understanding treatment including alternatives and adjuvant therapy” is a cluster that goes through almost the entire journey, while the “Daily activities while living with colorectal cancer,” is more in the home context and starts later in the follow-up.

**Table 1.** Overview of the clusters derived from the topics and their keywords.

Clusters andtopics, (n, %)^a^ (N=212,107)		Top 20 keywords	Number of posts^b^, n
Cluster 1: Experience around medical professionals’ opinion (9756, 4.6%)			
Doubts about treatment options from medical professionals		‘doctor’, ‘oncologist’, ‘surgeon’, ‘told’, ‘patient’, ‘system’, ‘cell’, ‘wrong’, ‘reason’, ‘medical’, ‘asked’, ‘office’, ‘immune’, ‘trust’, ‘kill’, ‘testing’, ‘medicine’, ‘appointment’, ‘clinic’, ‘recommend’	5043
Suggestion to look for a second medical opinion		‘care’, ‘second’, ‘opinion’, ‘ask’, ‘port’, ‘onc’, ‘put’, ‘taken’, ‘nurse’, ‘center’, ‘forget’, ‘top’, ‘first’, ‘team’, ‘comfortable’, ‘third’, ‘schedule’, ‘getting’, ‘question’, ‘hospital’	4713
Cluster 2: Understanding treatment including alternatives and adjuvant therapy (31,577, 14.9%)			
Patients share their research about alternative therapy options from websites and articles		‘answer’, ‘information’, ‘might’, ‘research’, ‘alternative’, ‘therapy’, ‘study’, ‘perhaps’, ‘available’, ‘patient’, ‘consider’, ‘website’, ‘based’, ‘benefit’, ‘article’, ‘option’, ‘approach’, ‘internet’, ‘current’, ‘specific’	7854
Making treatment decisions for the future with regard to the best outcome and path		‘best’, ‘wish’, ‘wishing’, ‘decision’, ‘health’, ‘possible’, ‘whatever’, ‘future’, ‘choice’, ‘outcome’, ‘decide’, ‘path’, ‘upcoming’, ‘regard’, ‘situation’, ‘advocate’, ‘choose’, ‘action’, ‘simply’, ‘circumstance’	4759
Listing type, side effect, regimen, and effectiveness of drugs		‘side’, ‘effect’, ‘drug’, ‘folfox’, ‘round’, ‘cycle’, ‘dose’, ‘vitamin’, ‘week’, ‘oxaliplatin’, ‘avastin’, ‘reaction’, ‘irinotecan’, ‘affect’, ‘rash’, ‘onc’, ‘cell’, ‘regimen’, ’session’, ‘effective’	8768
Sharing experience on using traditional Chinese medicine to manage health		‘well’, ‘known’, ‘fairly’, ‘usual’, ‘responded’, ‘recall’, ‘crc’, ‘chinese’, ‘handling’, ‘version’, ‘correctly’, ‘content’, ‘deserved’, ‘treating’, ‘referring’, ‘nicely’, ‘handled’, ’spelling’, ‘managing’, ‘tolerated’	3610
Share experiences and recommendations for supplements and medication		‘congratulation’, ‘definitely’, ‘tried’, ‘experience’, ‘mentioned’, ’suggestion’, ‘type’, ‘helped’, ‘mention’, ‘medication’, ‘interested’, ’speak’, ‘form’, ‘help’, ’suggest’, ‘using’, ’supplement’, ’suggested’, ‘detail’, ‘order’	2854
Sharing information about and experiences regarding clinical trials		‘new’, ‘find’, ‘look’, ‘looking’, ‘awesome’, ‘forward’, ‘trial’, ’start’, ‘working’, ’step’, ‘move’, ‘hearing’, ‘hard’, ‘pic’, ‘yet’, ‘clinical’, ‘finding’, ‘meeting’, ‘hopeful’, ‘totally’	3732
Cluster 3: Surgery experience (9540, 4.5%)			
Sharing experience around radiofrequency ablation for the liver		‘congrats’, ‘procedure’, ’success’, ‘huge’, ‘liver’, ‘proud’, ’shrink’, ‘treat’, ‘entire’, ‘tumor’, ‘ablation’, ‘option’, ‘rfa’, ‘candidate’, ‘location’, ’status’, ’shrinkage’, ‘inoperable’, ’surgical’, ‘afterwards’	4979
Sharing negative experience about resection in liver and lung		‘day’, ‘liver’, ‘resection’, ‘mets’, ‘every’, ‘met’, ‘pump’, ‘lung’, ‘ahead’, ‘week’, ‘couple’, ‘following’, ‘open’, ‘two’, ‘operation’, ‘followed’, ‘throughout’, ’single’, ‘complication’, ’section’	4561
Cluster 4: Experience regarding the test results (including being worried and confused) ( 29,984, 14.1%)			
Sharing negative emotions and experiences to live with colorectal cancer (CRC): overwhelmed, confused and scared, especially for the tests and screenings		‘like’, ’sound’, ‘wonderful’, ‘plan’, ‘place’, ‘picture’, ’show’, ‘meet’, ‘report’, ‘looked’, ‘absolutely’, ‘really’, ‘pick’, ‘button’, ‘familiar’, ‘machine’, ‘pretty’, ’smart’, ’silly’, ‘compare’	3873
Being worried about upcoming scans and results		‘great’, ‘result’, ‘idea’, ’scan’, ‘number’, ‘option’, ‘curious’, ‘present’, ‘follow’, ‘considering’, ‘treated’, ‘recurrence’, ‘appendix’, ‘initial’, ‘cea’, ‘mop’, ‘peritoneal’, ‘assuming’, ‘base’, ‘reliable’	3210
Share their outcomes (clear or not) from scanning and caring about how frequently they scan		‘year’, ‘month’, ‘ago’, ‘clear’, ‘last’, ‘three’, ‘two’, ‘free’, ’scan’, ‘colonoscopy’, ‘end’, ‘past’, ‘every’, ‘four’, ‘clean’, ‘date’, ’six’, ’safe’, ‘behind’, ‘later’	4293
Describing a stressful experience in a blood test; worry about the numbers in the result		‘everyone’, ‘word’, ‘blood’, ‘test’, ‘kind’, ‘wait’, ‘worry’, ‘fine’, ‘check’, ‘waiting’, ‘count’, ‘normal’, ‘wanted’, ‘level’, ‘concern’, ‘checked’, ‘else’, ‘low’, ‘high’, ‘appointment’	5048
Being worried and confused about odd scan results in liver, lung, and lymph		’scan’, ‘tumor’, ‘liver’, ‘node’, ‘lung’, ‘removed’, ‘radiation’, ’stage’, ‘lymph’, ’spot’, ’showed’, ’spread’, ’small’, ‘pet’, ‘biopsy’, ‘remove’, ‘mets’, ’surgeon’, ‘recurrence’, ‘week’	13,560
Cluster 5: Experience with side effects (26,152, 12.3%)			
Sharing their experience on managing the side effects of treatment		‘cold’, ‘nausea’, ‘taking’, ‘help’, ‘water’, ‘drink’, ‘warm’, ‘pill’, ‘infusion’, ’sleep’, ‘mouth’, ’sore’, ‘med’, ‘oxy’, ‘gave’, ‘fatigue’, ‘mum’, ‘nasty’, ‘appetite’, ’sleeping’	8341
Negative feelings of hair loss due to cancer treatments		’stuff’, ‘hair’, ‘funny’, ‘hate’, ‘bad’, ’suck’, ‘lose’, ‘cut’, ‘fall’, ‘wear’, ‘crap’, ‘lost’, ‘really’, ‘made’, ’stand’, ‘head’, ‘weird’, ‘losing’, ’strange’, ‘air’	5122
Feeling uncomfortable due to the obstipation		‘pain’, ‘control’, ‘nothing’, ‘relief’, ‘med’, ‘bowel’, ‘issue’, ‘breath’, ‘walking’, ‘walk’, ’scar’, ‘deep’, ‘kidney’, ‘causing’, ‘blockage’, ‘problem’, ‘intestine’, ‘tube’, ‘hospital’, ‘hernia’	6414
Experiencing pain and numbness due to neuropathy		‘hand’, ‘foot’, ‘hurt’, ‘neuropathy’, ‘bone’, ‘worse’, ‘arm’, ‘pain’, ‘caused’, ‘leg’, ‘left’, ‘painful’, ‘problem’, ‘experienced’, ‘damage’, ‘nerve’, ‘cause’, ‘related’, ’shoulder’, ’symptom’	6275
Cluster 6: Confusion with insurance (7912, 3.7%)			
Confusion about insurance coverage		‘insurance’, ‘really’, ’sent’, ‘email’, ‘needed’, ‘pay’, ‘company’, ‘money’, ’state’, ‘phone’, ‘cost’, ‘card’, ‘mail’, ‘received’, ‘hospital’, ‘address’, ‘cover’, ‘attention’, ‘letter’, ’service’	7912
Cluster 7: Experience during recovery phase (8632, 4.1%)			
Describing negative experience of repeated visits to the hospital		‘time’, ‘took’, ‘full’, ‘week’, ’short’, ‘last’, ’shot’, ‘infection’, ‘heal’, ‘ended’, ‘went’, ‘due’, ‘drop’, ‘period’, ’several’, ’stopped’, ‘hospital’, ’started’, ‘kept’, ‘taking’	5010
Staying positive and making life adjustments to their cancer circumstances during the recovery phase		‘way’, ‘positive’, ’sending’, ‘coming’, ‘along’, ‘vibe’, ‘healing’, ‘energy’, ‘half’, ‘begin’, ‘rid’, ‘improve’, ‘ton’, ‘complete’, ‘outlook’, ‘faster’, ‘headed’, ‘toward’, ‘improved’, ‘adjustment’	3622
Cluster 8: Mindset-related attitude living with CRC (26,379, 12.4%)			
Difficulties to adjust and adapt to their lives with CRC		‘going’, ‘time’, ’sometimes’, ‘enough’, ‘trying’, ‘probably’, ‘part’, ‘really’, ‘actually’, ‘course’, ‘change’, ‘either’, ‘pretty’, ‘different’, ‘body’, ‘point’, ‘never’, ‘hard’, ‘anyway’, ‘whole’	6332
Sharing how patients can be resilient and positive		‘always’, ’stay’, ‘remember’, ‘away’, ‘week’, ‘next’, ‘cry’, ‘laugh’, ’sense’, ’strong’, ‘forever’, ‘matter’, ‘extra’, ‘humor’, ’staying’, ‘never’, ‘whenever’, ‘hero’, ’sweetie’, ‘corner’	4449
Sharing their feelings: sick, tired, weak, and bad		‘feel’, ‘better’, ‘feeling’, ’soon’, ‘hopefully’, ‘getting’, ’sick’, ‘felt’, ‘tired’, ‘real’, ‘making’, ‘really’, ’starting’, ‘bad’, ’stronger’, ‘gotten’, ’start’, ‘expected’, ‘weak’, ‘normal’	4520
Sharing their positive philosophical thoughts about living with cancer		‘life’, ‘live’, ‘mean’, ‘understand’, ‘people’, ‘important’, ‘living’, ‘world’, ‘fear’, ‘time’, ‘become’, ‘realize’, ‘death’, ‘die’, ‘quality’, ‘learn’, ‘focus’, ‘illness’, ‘save’, ‘allow’	5451
Survivors sharing their attitudes towards living with cancer along with survival rate		’story’, ‘old’, ’survivor’, ‘disease’, ‘folk’, ’site’, ‘year’, ‘member’, ‘cure’, ‘people’, ‘recently’, ‘personal’, ‘passed’, ‘woman’, ‘remission’, ‘given’, ’survival’, ‘grateful’, ‘alive’, ‘lived’	5627
Cluster 9: Interaction with family and friends (23,393, 11%)			
Family members’ emotional struggle about having a cancer patient in their family		‘husband’, ‘heart’, ‘face’, ‘eye’, ‘hold’, ‘head’, ‘tear’, ‘dog’, ’stop’, ‘brought’, ‘hell’, ‘front’, ‘putting’, ‘child’, ’soul’, ‘biggest’, ‘attack’, ‘mad’, ‘horse’, ‘men’	4798
Experience regarding relationships with friends while having cancer		‘got’, ‘friend’, ‘heard’, ‘name’, ‘room’, ’semi’, ‘never’, ‘bunch’, ‘forgot’, ‘hot’, ‘blue’, ‘chat’, ’surprise’, ‘joke’, ‘facebook’, ‘picked’, ‘neighbor’, ‘girlfriend’, ‘Canadian’, ‘everywhere’	4062
Sharing changes in their family relationship due to cancer journey		‘thinking’, ‘today’, ‘family’, ‘anyone’, ‘call’, ‘wondering’, ’sister’, ‘called’, ‘brother’, ‘visit’, ‘yesterday’, ‘talk’, ‘close’, ‘else’, ’sign’, ‘talking’, ‘law’, ‘wanted’, ‘concerned’, ‘appt’	3189
Being worried about their family members and seeking information on family history (eg, genetic testing)		‘mom’, ‘dad’, ‘agree’, ‘mother’, ‘age’, ‘never’, ‘worried’, ‘father’, ‘stage’, ‘breast’, ‘symptom’, ‘died’, ‘diagnosed’, ‘yr’, ‘turned’, ‘happen’, ‘parent’, ‘told’, ‘tested’, ‘child’	5273
Arguing the importance to spend moments with family members during the cancer journey		‘night’, ‘wife’, ‘daughter’, ‘kid’, ‘hour’, ‘beautiful’, ‘morning’, ‘together’, ‘friday’, ‘every’, ‘minute’, ‘day’, ‘moment’, ‘late’, ‘last’, ‘bed’, ‘home’, ‘thursday’, ‘gift’, ‘time’	6071
Cluster 10: Daily activities while living with the CRC (38,782, 18.3%)			
Sharing suggestions on diets focused on balanced meals and healthy alternatives		‘eat’, ‘food’, ‘cheer’, ‘diet’, ‘eating’, ‘juice’, ‘sugar’, ‘juicing’, ‘favorite’, ‘fruit’, ‘glass’, ‘perfect’, ‘dream’, ‘green’, ‘drink’, ‘red’, ‘wine’, ‘meat’, ‘coffee’, ‘heck’	9910
Suggesting how to take good care of a stoma.		‘bag’, ‘problem’, ‘add’, ‘colostomy’, ‘used’, ‘daily’, ‘ostomy’, ‘twice’, ‘radiation’, ‘reversal’, ‘bathroom’, ‘ileostomy’, ‘skin’, ‘diarrhea’, ‘prep’, ‘stool’, ‘stoma’, ‘rectum’, ‘careful’, ‘bowel’	8418
Sharing experience on being fit again, caring about weight control		‘back’, ‘came’, ‘weight’, ‘exercise’, ‘return’, ‘set’, ‘running’, ‘self’, ‘track’, ‘pound’, ‘door’, ‘gain’, ‘lost’, ‘lb’, ‘fit’, ‘buzzard’, ‘sit’, ‘key’, ‘floor’, ‘put’	5460
Sharing ways to stay in a positive mood through planning for distractions		‘going’, ‘enjoy’, ‘weekend’, ‘fun’, ‘trip’, ‘ready’, ‘tonight’, ‘vacation’, ‘game’, ‘watch’, ‘party’, ‘drive’, ‘house’, ‘weather’, ‘excited’, ‘car’, ‘town’, ‘near’, ‘planning’, ‘movie’	6949
Celebrating anniversaries and birthdays for patients as a big milestone of their lives		‘happy’, ‘birthday’, ‘dance’, ‘holiday’, ‘healthy’, ‘yea’, ‘anniversary’, ‘thanksgiving’, ‘naked’, ‘celebrating’, ‘celebration’, ‘filled’, ‘happiness’, ‘raise’, ‘dancing’, ‘cake’, ‘spongebob’, ‘ending’, ‘celebrated’, ‘scouty’	8045

a Number of topics in each cluster.

b Number of posts related to the topic group.

## Discussion

### Principal Findings

This study delves into the daily experiences of patients using data from a web-based platform, applying topic modeling as a tool to efficiently analyze and interpret large volumes of patient-driven daily life experience data. We analyze patient experiences (eg, struggles, tips, and coping strategies) reported on a platform to better understand patient needs. This understanding of patients’ needs has positive potential for future care path development such as information provision or psychosocial support in remote patient monitoring. The use of machine learning techniques in analyzing qualitative data holds significant promise to improve various aspects of health care. This includes optimizing patient care, by using advanced methods such as using symptom checkers to guide patients to the most suitable care journeys and support disease diagnosis. This approach will ultimately lead to patient-centered care to support and empower patients.

The identified clusters in this study align with findings from previous qualitative and survey research, emphasizing the potential to optimize the use of machine learning in qualitative studies of health care in the years to come [4,23]. Based on our analysis, it becomes evident that patients exhibit a notable interest in actively participating in discussions with other CRC survivors to exchange experiences regarding the daily challenges of living with CRC. This underscores the crucial role of social support and the dissemination of pragmatic, day-to-day coping strategies within the broader patient community. This theme aligns with observations from a systematic review detailing the experiences of CRC survivors, where the significance of coping and addressing functional limitations emerged as a central theme [23].

The cluster with the highest share of posts (cluster 10; 38,782, 18.3%) revolves around the challenges associated with resuming daily activities while living with CRC. This substantial volume of posts highlights the significant impact of this particular aspect on the experiences of CRC survivors, as well as the needed change in patients’ mindset (cluster 8; 26,379/212,1087, 12.4%). The systematic review of survivorship experiences further supports these findings [23]. The alignment between these dominant themes in the posts and the insights from the qualitative literature underscores the robustness of our findings [24]. These findings emphasize the importance of integrating comprehensive support mechanisms to facilitate the successful reintegration of patients into their daily lives.

On the platform, patients frequently engage in discussions centered around understanding treatment options (cluster 2; 31,577, 14.9%), highlighting its significance as a key topic. This observation aligns with the qualitative study conducted by van Deursen et al [4], where participants similarly emphasized the need for more information regarding their treatment. It is noteworthy that patients on the platform also frequently express concerns regarding test results (cluster 4; 29,984, 14.1%). This anxiety and fear of recurrence are recognized as common concerns among cancer survivors [25], highlighting the potential to offer valuable support in addressing this. Additionally, the positive outcomes associated with the interaction with family, as reported in the systematic review [23], further underscore the importance of familial support within the context of patient experiences in our findings [26].

A novel finding from our analysis was the open expression of patients’ sentiments regarding the opinions of medical professionals (cluster 1; 9756, 4.6%). Not completely surprising as this is possibly due to the reluctance of patients to express negative feelings in a setting where researchers are present, such as focus groups or interviews. Furthermore, qualitative studies primarily concentrate on the follow-up phase, excluding discussions on the experiences of surgery and the subsequent recovery phase. These aspects might provide valuable insights into the comprehensive journey of patients.

### The Added Value of Machine Learning to Support the Understanding of Patient Experiences

To reflect on our approach, topic modeling allows extraction from a vast pool of large-scale patient data. The alignment of our study with existing literature is indeed promising. Additionally, topic modeling offers a novel approach by sparing patients the burden of active participation in qualitative studies. Rather than focusing on a single aspect, as is often the case in conventional studies, this method presents a broader spectrum of patient experiences beyond specific questions from researchers. Notably, topic modeling relies on patient-driven data, in contrast to a physician-driven approach. This method’s replicability allows seamless comparisons between various medical conditions. This not only contributes to a more extensive comprehension of diverse experiences but also facilitates the identification of commonalities and distinctions of different medical conditions. For example, we explored the present method with patients with pulmonary fibrosis and found comparable experiences to those with CRC, their increased focus on treatment options highlights the distinctive challenges inherent to their specific condition (unpublished data). Given the large data sets on forums, we can quantify patients’ experiences with the implementation of topic modeling. This complements the challenges of focus groups and interviews, where the ability to quantify and prioritize findings is limited. As patients’ experiences change throughout their journey, the simplicity of using topic modeling remains a flexible and efficient approach for understanding patients’ experiences and priorities in the era of remote monitoring [27]. It offers a convenient and adaptable method, without burdening patients [28].

### Limitations

Using stories from patients who prefer to share their experiences on a digital platform introduces a risk of selection bias. It is essential to address the potential overexpression of emotion in our findings as the platform primarily captures narratives from individuals who are willing to share their experiences on the web. However, it should be recognized that these individuals represent the intended user group for new care path developments such as remote monitoring support approaches. Patients who are not feeling well, patients who do not have access to the internet, or reserved patients may be less inclined to engage in web-based discussions and might be underrepresented [29]. To complement this, in previous research, we conducted interviews with vulnerable patient populations to ensure their perspectives were included in our understanding of patient experiences [9]. Similar themes such as the need for information provision, concerns about test results, and challenges in reintegrating into daily life were found in this study. Additionally, our approach demands a significant investment of human labor and time to interpret the meaning of topics after generating groups of topics. Thus, exploring another artificial intelligence technique to interpret patient experiences is a promising direction. As an example, a large language model, a pretrained model, can be used to interpret and summarize large quantity textual data [30]. The use of machine learning, even in the part where we used human experts’ knowledge, is a promising area to labor-effectively understand the patients’ experience while having the least safeguarding support of human validation. Another limitation is the lack of direct involvement of patients with CRC in the co-design or review of the map. We plan to address this in the future as this can provide valuable insights. However, the primary purpose of this method is to enhance the methodology while minimizing the burden on patients. To overcome this limitation, we sought input from highly experienced medical professionals’ perspectives. Three of these experts possessed more than 20 years of medical and research experience, specifically with patients with CRC.

In future research, efforts should be directed towards further refining machine learning techniques with the ultimate aim of minimizing or eliminating the need for human involvement, as this will enable more frequent monitoring of patient experiences and thereby facilitate responsiveness to those experiences. Addressing the nuanced interpretation of emotions in patients remains a challenge for artificial intelligence systems. A promising direction is the automatic interpretation of patient-reported outcome measures using machine learning and NLP. Patient-reported outcome measures offer structured data that can improve AI’s ability to understand emotional nuances. This approach aims to improve remote patient monitoring, reduce the burden on health care professionals, and identify those who face psychosocial challenges [31]. This approach not only empowers patients in managing their health but also leads to a proactive and personalized approach.

In conclusion, topic modeling and the use of patient forum data offer a robust and efficient approach to understanding patients’ experiences in their daily lives. This approach reveals the challenges patients encounter in their daily life such as getting back to daily activities and the need for understanding their treatment. This study not only provides a comprehensive overview of patient experiences through web-based platforms but also highlights the potential for improving patient monitoring systems. This machine learning technique of identifying patient experiences contributes to a more efficient way of building value-based health care. By integrating these insights into the development of remote monitoring solutions, a patient-centered approach can be created that not only addresses medical concerns but also caters to the broader spectrum of challenges individuals face in their daily lives.

## Supplementary material

10.2196/58834 Multimedia Appendix 1 Consolidated reporting guidelines for prognostic and diagnostic machine learning modeling studies

## Abbreviations

CRC: colorectal cancer
NMF: negative matrix factorization
