# Supplementary material for: A Machine Learning Approach Using Topic Modeling to Identify and Assess Experiences of Patients With Colorectal Cancer: Explorative Study
Source: JMIR Cancer. 2025 Jan 27;11:e58834. doi: 10.2196/58834 (PMC11790180; doi:10.2196/58834)
Supplement: Multimedia Appendix 1 [file cancer-v11-e58834-s001.pdf]

Consolidated reporting guidelines for prognostic and diagnostic machine learning modeling studies

Our study is not strictly a prognostic or diagnostic study as defined by the 'Consolidated Reporting Guidelines for prognostic and diagnostic machine learning modeling studies'. Our research is exploratory in nature. While we adhere to the relevant aspects of the guideline, certain elements may not fully apply to our study's objectives and methodology.

Multimedia Appendix 1: Author Checklist

The following is the reporting checklist. A response should indicate whether the particular item is documented in the study. If the response to an item is Y then the location in the article should be provided (e.g., section number), and if the response is N or NA then some reasoning should be provided.

#	Item	Y	N	NA	Location / Reasoning
**Study Details**					
1.1	*The medical/clinical task of interest*	x			Introduction, p2
1.2	*The research question*	x			Introduction, p2
1.3	*Current medical/clinical practice*	x			Introduction, p2
1.4	*The known predictors and confounders to what is being predicted / diagnosed*	x			Introduction, p2
1.5	*The overall study design*	x			Introduction, p2
1.6	*The medical institutional setting(s)*	x			Methods, p3
1.7	*The target patient population*	x			Introduction, p2
1.8	*The intended use of the ML model*	x			Introduction, p2
1.9	*Existing model performance benchmarks for this task*		x		
1.10	*Ethical and other regulatory approvals obtained*	x			Methods, p3
**The Data**					
2.1	*Inclusion / exclusion criteria for the patient cohort*	x			Methods, p3
2.2	*Methods of data collection*	x			Methods, p3
2.3	*Bias introduced due to the method of data collection used*	x			Limitations, p13
2.4	*Data characteristics*	x			Results, p4
2.5	*Methods of data transformations and preprocessing applied*	x			Methods, p3
2.6	*Known quality issues with the data*	x			Limitations, p13
2.7	*Sample size calculation*			x	We used all content from the forum

2.8	*Data Availability*	x			Open source, p3
**Methodology**					
3.1	*Strategies for handling missing data*			x	Our data source consists of user-generated forum posts from the Cancer Survivors Network. Since these posts are inherently complete as individual data points, the concept of missing data as it applies to structured datasets does not apply to our analysis.
3.2	*Strategies for addressing class imbalance*			x	Our study involves unsupervised topic modeling, which does not classify data into predefined categories but rather identifies latent topics within the text data. Therefore, class imbalance is not a relevant issue.
3.3	*Strategies for reducing dimensionality of data*	x			Methods, p3
3.4	*Strategies for handling outliers*			x	In our context of qualitative data analysis, unusual posts are part of the richness of the patient experience and are not treated as outliers to be removed but as integral parts of the dataset.
3.5	*Strategies for data augmentation*			x	Our study relies on naturally occurring forum posts, and artificially generating posts would not accurately reflect genuine patient experiences, thus data augmentation is not

					applicable.
3.6	*Strategies for model pre-training*			x	Our topic modeling approach does not require pre-training, as it is directly applied to the text data from the CRC patient forum to discover topics.
3.7	*The rationale for selecting the machine learning algorithm*	x			Methods, P3
3.8	*The method of evaluating model performance during training*	x			Methods, p4
3.9	*The method used for hyperparameter tuning*			x	We did not apply hyperparameter tuning for the responses generated in our study. Instead, we used a pre-trained language model, GPT-3.5, and employed prompt engineering to ensure the context of our topic results was accurately interpreted. Key terms from the topic results were included in the model's requests to generate relevant responses. These responses were subsequently evaluated by medical experts for their relevance in describing patient experiences. Hyperparameter tuning would have required fine-tuning the model on our specific topic results, which was not done due to its exploratory nature and the emphasis on gaining initial qualitative insights rather than

					optimizing model performance.
3.10	*Model's output adjustments*	x			Methods, p4
**Evaluation**					
4.1	*Performance metrics used to evaluate the model*	x			Methods, p5/6<br>Results p7/8
4.2	*The cost or consequence of errors*	x			Discussion, p13
4.3	*The results of internal validation*	x			Page 4 methods
4.4	*The final model hyperparameters*	x			Results p7/8
4.5	*Model evaluation on an external dataset*			x	Model evaluation on an external dataset was not conducted in this study because the primary focus was on exploring and mapping CRC patient experiences within a specific dataset (Cancer Survivors Network USA).
4.6	*Characteristics relevant for detecting data shift and drift*			x	Since our study did not involve time-dependent predictions or real-time data analysis, detecting shifts or drifts in data was not pertinent to our research objectives.
**Explainability and Transparency**					
5.1	*The most important features and how they relate to the outcome(s)*	x			Conclusion, p13/14
5.2	*Plausibility of model outputs*	x			Conclusion, p13/14
5.3	*Interpretation of model's results by an end-user*	x			Conclusion, p13/14
